# Subtyping of Internalizing and Externalizing Behaviors in Japanese Community-Based Children: A Latent Class Analysis and Association with Family Activities

**DOI:** 10.3390/children9020210

**Published:** 2022-02-06

**Authors:** Xiang Li, Yan-Tong Zhu, Dan-Dan Jiao, Yuko Sawada, Emiko Tanaka, Taeko Watanabe, Etsuko Tomisaki, Zhu Zhu, Ammara Ajmal, Munenori Matsumoto, Jin-Rui Zhang, Alpona Afsari Banu, Yang Liu, Ming-Yu Cui, Yolanda Graça, Yan-Lin Wang, Mei-Ling Qian, Tokie Anme

**Affiliations:** 1 School of Comprehensive Human Science, University of Tsukuba, Tsukuba 3058577, Japan; lixiangdufl@gmail.com (X.L.); zyt199431@gmail.com (Y.-T.Z.); jdd201304@163.com (D.-D.J.); zhuzhu881231@yahoo.co.jp (Z.Z.); ammara.ajmal6@gmail.com (A.A.); 11MN023w9Y-ts@slcn.ac.jp (M.M.); jinrui970308@yahoo.co.jp (J.-R.Z.); afsarialpona94@hotmail.com (A.A.B.); dataerliu@yahoo.co.jp (Y.L.); cuimingyujiahua@163.com (M.-Y.C.); yolandagraca28@gmail.com (Y.G.); wangyanlin202004@163.com (Y.-L.W.); qianmeiling2020@126.com (M.-L.Q.); 2 Faculty of Health Medicine, Morinomiya University of Medical Sciences, Osaka 5598611, Japan; y-sawada@morinomiya-u.ac.jp; 3 Faculty of Nursing, Musashino University, Tokyo 2028585, Japan; warakott@gmail.com; 4 Faculty of Nursing, Shukutoku University, Chiba 2608701, Japan; tae@fureai.or.jp; 5 Faculty of Nursing, Keio University, Tokyo 1088345, Japan; ettsukot@gmail.com; 6 Faculty of Medicine, University of Tsukuba, Tsukuba 3058577, Japan

**Keywords:** internalizing and externalizing behaviors, family activities, community-based, Japanese children, latent class analysis

## Abstract

Externalizing and internalizing behavioral problems occurs at a high rate among children. However, this has rarely been examined among Japanese children using a person-oriented method. Hence, this study aims to explore its subtypes and clarify their association with family-based group activities. We conducted a cross-sectional survey in a typical community-based suburban area for all families with primary school children in Japan. We investigated children’s internalizing and externalizing behaviors based on the Japanese version of the Strengths and Difficulties Questionnaire (SDQ), and different types of activities that family members frequently engaged in. Data from 206 families were collected and used for the analysis. The subtypes were explored using latent class analysis (LCA). The relationship between family activities and latent class membership was analyzed using a logistic regression model. Moreover, three latent class models and their probabilities were identified, namely, risk group (31.3%), moderate group (44.9%), and normal group (23.8%). Frequent family activities including play sports, traveling or hiking, watching TV and communicating, cooking or making a dessert, and doing housework, which were significantly related to the normal group. These results would add evidence to potential types of children’s behavioral problems and preventive childcare practices needed in the primary gate of families.

## 1. Introduction

Externalizing behaviors are defined as a range of disruptive and dysregulated behaviors, including aggression, conduct problems, delinquent behavior, oppositionality, hyperactivity, and attention problems. Meanwhile, internalizing behaviors are characterized as behavioral patterns directed inward toward oneself, including anxiety, fear, sadness/depression, social withdrawal, and somatic complaints [1,2,3]. Both behavioral problems co-occur at a high rate among children, and this simultaneous occurrence may be more common than either of them occurring on its own [4,5,6,7,8,9,10]. Recently, co-occurring emotional and behavioral problems have been reported, and this may be further associated with autism spectrum disorder among children [8,9,10]. The comorbidity of both behavioral problems has been related to higher levels of impairment and a higher risk of developing psychiatric disorders in adulthood, criminal offenses, and suicide [11].

The co-occurrence of psychiatric disorders across similar and different domains can be termed as homogeneous and heterogeneous comorbidity [12]. The heterogeneous comorbidity of externalizing and internalizing problems could be concluded as “bad things are related to other bad things” [13]. Over the last decade, many studies focusing on the structure of internalizing and externalizing psychopathology have been conducted using the variable-oriented method. Several existing studies used pre-specified cut-off points on dimensional internalizing and externalizing symptom scales as the classification method [1,11,14]. A homogeneous group will be categorized among children if their scores are above the cut-off point. Although this method could capture information about the relationship between the variables-of-interest for the overall sample, it did not consider the patterns of diversity among children’s characteristics and limited the distinct features of heterogeneity within a given sample in subgroups [15,16]. Thus, person-based methods, such as latent class analysis (LCA), can clarify the heterogeneous groups of both behavioral disorders in children with similar patterns of psychopathology [16,17]. LCA is a statistical technique commonly used in mental health research to identify the subtyping of disorders [15]. 

A classical instrument, called the Strengths and Difficulties Questionnaire (SDQ) [18] has been widely used in multiple countries, both in community and clinical populations. It can detect children’s internalizing and externalizing behavioral problems and covers the most prevalent area of psychopathology in children [4]. Although studies using SDQ have been widely conducted in the last decade to clarify the relationship between risk or protective factors and children’s internalizing and externalizing behavioral problems, subtyping of both behavioral problems regarding SDQ has been explored to a limited extent using LCA. Using LCA for SDQ, three groups were identified among community-based adolescents in China, including the low, middle and high levels of co-occurring [4]. Three groups were also found among the left-behind children under 18 years old in China, including maladjustment, behavior impulse, and basically adapted groups [19]. As for primary school age, five groups were identified in a sample of Spanish children aged 7–12 years old, including internalizing, externalizing, high difficulties, well-adjusted, and hyperactive groups [20]. Together, previous studies identified the heterogeneity of internalizing and externalizing behaviors among children. Knowing the different subtypes may help provide information about what specific types should be addressed for adequate prevention or intervention in clinical settings [4,20]. However, the subtyping of both behaviors varied greatly according to characteristics of children [4], which is needed to explore the potential types with diverse samples to extend the evidence for prevention strategies. Moreover, children in community samples have shown a high co-occurrence of both behavioral problems and tend to experience more severe symptoms [1,13,20], while the subtyping of both behaviors among community-based children’s samples is still limited.

Subtyping among primary-school aged children is still limited in Asian countries. To our knowledge, no study has identified subtyping of internalizing and externalizing behaviors using a person-based method among Japanese children. Additionally, most previous studies did not consider factors related to subtyping. Those studies only examined demographic characteristics such as age, gender, and siblings [4,19,20]. Family is the first health avenue for children. Studies have shown that parents’ positive engagement with children in social activities is correlated with children’s general emotional and behavioral development [21,22]. A positive parent–child relationship can be a protective factor against children’s internalizing or externalizing behavioral problems [23,24], and this relationship can be encouraged by family activities, such as playing games and sharing meals [25,26]. Thus, we hypothesize that factors of family activities are also related to the latent class memberships of children’s internalizing or externalizing behaviors. Knowing the types of family activity may help support institutions provide information to family to empower and support their children’s emotional and behavioral development. 

In summary, we aim to (1) clarify the potential types of children’s internalizing and externalizing behaviors using a Japanese community-based sample, and (2) examine the relationship between different types of family activities and identify subtyping groups.

## 2. Methods

### 2.1. Design and Participants

This cross-sectional study is part of an ongoing longitudinal project called Community Empowerment and Care (CEC) for well-being and healthy longevity [24,27,28]. It was conducted in a typical community in a suburban area (population: 4539) near a large city in central Japan every three years. All residents were invited and agreed to participate. Drop-off/pick-up surveys and mailed surveys were conducted using self-administered questionnaires. Interviews were also conducted by the research staff with the participants who needed help in responding to the questionnaire. This longitudinal study is part of a health census survey of the community, conducted every three years, in co-operation with the local government. The longitudinal study was initiated in 1991 and aimed towards assessing risk factors associated with well-being and to improve the quality of life of all residents, including children, teenagers, adults, and elderly. No incentive for participation was provided. Only the data from children were used in the present study. A total of 216 families with primary school children in this community were recruited in 2017. A total of 206 paper questionnaires were completely answered by the parents. With regard to missing data, 10 participants were missing in terms of demographic characteristics. Finally, data from 206 parents (utilization rate: 95.4%) were included in the analysis. Among the families, 109 (52.9%) were boys. The mean age of these children was 8.45 (±1.70) years.

### 2.2. Ethical Consideration

This study was approved by the Ethics Committee of the University of Tsukuba, Japan (1331-3).

### 2.3. Procedure

We recruited all families with primary school children from a suburban community-based village in 2017. A set of self-report questionnaires was used to collect data on the SDQ from the parents. To explore the factors associated with community health and longevity, we collected data every three years from all residents.

### 2.4. Measures

The Strengths and Difficulties Questionnaire (SDQ) [18] was used to evaluate children’s internalizing and externalizing behaviors. The SDQ includes 25 items divided into five subscales of five items each, including conduct problems, hyperactivity/inattention, emotional symptoms, peer relationship problems, and pro-social behavior. These subscales can be divided into the following two scales: internalizing (emotional symptoms and peer relationship problems) and externalizing behaviors (conduct problems and hyperactivity/inattention) [29]. Each question included three choices (normal/borderline/abnormal). The Japanese version of the SDQ has been proven to be a reliable and useful instrument [30,31]. The cut-off point for each item was based on normal versus borderline/abnormal according to a rural community-based study conducted in Japan [24]. A previous study indicated the internal consistency coefficient among a countrywide sample of Japanese schoolchildren is 0.81 [30]. In this study, the internal consistency coefficient was 0.81, which showed a good level of reliability as in the previous study. Additionally, the internal consistency coefficients of internalizing and externalizing domains are 0.71 and 0.74, respectively. 

Nine activities were used to investigate the most frequent family activities of participants, namely, (1) eating outside, (2) playing sports, (3) traveling or hiking, (4) going to cinema or concert, (5) going shopping, (6) playing indoor games, (7) watching TV and communicate, (8) cooking or making a dessert, and (9) doing housework. For each question, a binary choice (yes/no) was used. Parents who selected “Yes” for each question were regarded as having done this kind of activity. The types of activities were summarized and set based on a family survey conducted by the Ministry of Health, Labour and Welfare [32].

There were five covariates used in this study, i.e., (1) age of children, (2) gender of children, (3) family structure, (4) siblings, and (5) disease. As for the disease, each parent was asked a question (e.g., Did your child get ill, requiring continuous treatment for more than two weeks in the last year?). Answers were recorded using a binary choice (Yes/No).

### 2.5. Data Analysis

First, we used a person-based method called latent class analysis (LCA) to explore the potential types of children’s internalizing and externalizing behaviors. LCA can empirically group participants based on represented response patterns across multiple potential factors instead of categorizing them based on cut-off scores [33], which can be defined through item probabilities and class proportions, or the sample proportion represented in each latent class [34]. Since no single statistical criterion identified the best-fitting LCA model, we used several fit indices to statistically determine the best model selection. The Bayesian information criterion (BIC), Akaike information criteria (AIC), sample-size-adjusted BIC (BIC), and corrected Akaike’s information criterion (CAIC) most often identify the best-fitting model. BIC and CAIC have the best performance in both small and large sample cases [35]; therefore, BIC was the most reliable measure [33,36]. Smaller values of these fit indices indicate a great model. Additionally, entropy is a standardized index of model-selection accuracy, with higher values indicating better classification of individuals into groups and classes that are clearly delineated from one another [36], where values around 0.8, are considered to have good classification [33]. Previous studies have shown that when entropy is <0.6, the classification error is more than 20%, and the entropy decreases with an increasing sample size [37,38]. With these, the model selection in this study was based on BIC indices, and entropy was maintained above 0.8 to ensure its accuracy. Each item was regarded as a categorical variable in the LCA model.

After group identification, we used the chi-square test to determine the relationship between covariates, family activities, and each latent class membership. Then, we used a multinomial logistic regression model to test the relationship between family activities (significant results in the chi-square test) and latent class membership of children’s internalizing and externalizing behaviors. Family activities and children’s internalizing and externalizing behaviors were the independent and dependent variables, respectively. Covariates were set according to the significant results from the chi-square test. All these variables were regarded as categorical variables in the chi-square test and multinomial logistic regression model. Statistical significance was set at *p* < 0.05. The LCA analysis was performed using SAS software (version 9.4. English). A logistic regression model was performed using SPSS software (version 27.0. English).

There are no empirically tested guidelines regarding the minimum sample size for the use of LCA. A previous study showed that more indicators and higher quality indicators corresponded to a lower parameter bias [39]. With N ≥ 200, at least six high-quality indicators should be used with or without a covariate [39]. With this, we think that the sample size and indicators fit the analysis.

## 3. Results

The demographic characteristics of the children and their families among the children in the study are shown in Table 1.

Table 2 shows the goodness-of-fit statistics for the number of latent classes. BIC has the lowest goodness-of-fit statistics for the 3-class model. Additionally, we found that BIC was relatively low in both 2-class and 3-class models. However, considering that there are three indicators (including AIC, BIC, and aBIC), the 3 class model is better than the 2-class model. Finally, we selected the 3-class model that indicated well-delineated classes with an entropy of 0.82.

**Figure 1:**
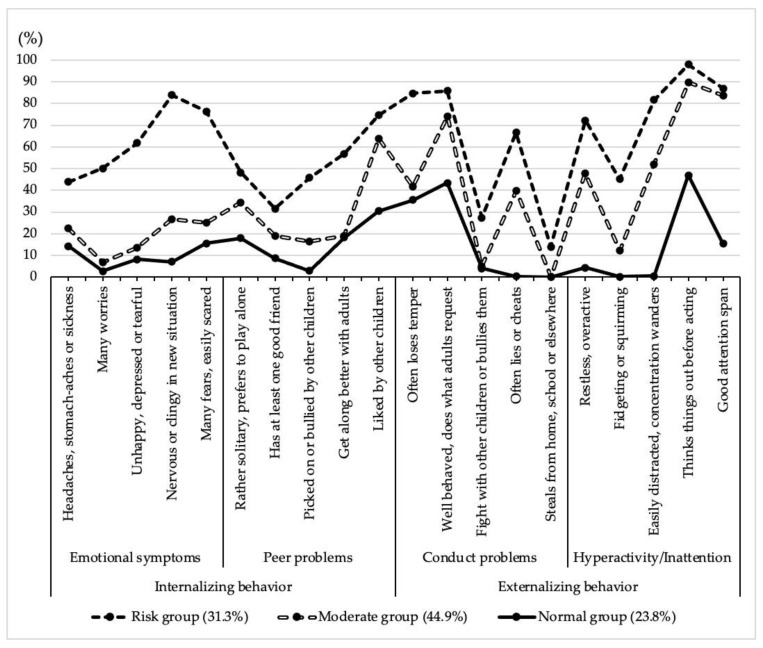
Item negative response probability plots for latent class analysis (LCA) of SDQ.

Table 3 and Figure 1 represent the probabilities of the three-class model. The feature of class 1 was that the children were most likely to experience the comorbidity of internalizing and externalizing behavioral problems. Most of the items’ probabilities in this class were higher than 40%. For class 2, the children were most likely to experience externalizing behavioral problems, while almost all internalizing indicators tended to be at the normal level. Class 3 comprised children with the lowest probability of internalizing and externalizing behavioral problems. Almost all items’ probabilities in this class were lower than 30%. Hence, we named the 3 classes as risk group, moderate group, and normal group. The class membership probabilities were as follows: 31.3% in the risk group, 44.9% in the moderate group, and 23.8% in the normal group.

Table 4 presents the results of the chi-squared test. We found that family members frequently engage in activities such as playing sports, traveling or hiking, watching TV and communicating, cooking or making desserts, and doing housework. These were related to children’s internalizing and externalizing behaviors.

Table 5 presents the results of the logistic regression model. From these models, we found that family members frequently engage in activities such as playing sports, traveling or hiking, watching TV and communicating, cooking or making desserts, and doing housework. These families were more likely to have the lowest probability of internalizing and externalizing behavioral problems. Children who engage in playing sports were less likely to be in the moderate (OR: 0.38; CI: 0.18–0.83) and risk groups (OR: 0.27; CI: 0.11–0.65). Children who engage in traveling or hiking activities were less likely to be in the risk group (OR: 0.32; CI: 0.12–0.83). Children who watched TV and communicated were less likely to be in the risk group (OR: 0.33; CI: 0.15–0.74). Children with a cook or dessert activity were less likely to be in the moderate group (OR: 0.32; CI: 0.13–0.74). Children who engaged in housework activities were less likely to be in the risk group (OR: 0.34; CI: 0.15–0.77). There were no significant results in eating outside, going to cinema or concert, going shopping and playing indoor games.

## 4. Discussion

This study provided a subtyping of children’s internalizing and externalizing behaviors according to a Japanese community-based sample of primary school children. We also presented correlations between family activities and latent class memberships of children’s internalizing and externalizing behaviors. We found evidence for different patterns of both behaviors in Japanese community-based samples. We identified that the comorbidity of both behavioral problems also existed in this sample population. The normal group showed low co-occurrence of both behaviors. The moderate group presented more hyperactivity/inattention problems with lying and behaved out of request. The risk group showed the highest probability of co-occurring internalizing and externalizing behavioral problems, especially emotional and hyperactivity/inattention problems. 

The probability of risk group in this study (31.3%) was similar to two other studies, including a Spanish children’s sample (34.2%) [20] and a Chinese children’s sample (32.0%) [19] which also identified the risk group of children’s internalizing and externalizing behaviors using the SDQ. However, the risk-group probability of another study conducted among Chinese community-based adolescents (aged 11–18 years old) was 19.8% [4]. A previous study revealed that the probability of a risk group among younger children was higher than for adolescents [20]. In our study, we also obtained a high probability of the risk group since the comorbidity of both behavioral problems has an early age onset, younger children indistinctively respond to stress, and score highly for symptoms of different syndromes [1,20]. 

The item probabilities of the risk group ranged from 40.0% to 80.0%. This means that the probability of showing both behavioral problems is moderate to high. In our study, the probabilities of items’ negative responses included “fight with other children or bullies them,” “steals from home, school or elsewhere”, and “has at least one good friend” were relatively low when compared to other items in the risk group. All of which were similar to the Spanish study [20]. 

A relatively high probability (44.9%) was found in the moderate group in our study, which is similar to the Chinese left-behind children’s study (41%) [19]. The Spanish children’s study showed that the total probability of the three moderate classes (three different classes between the risk group and normal group), named internalizing, externalizing, and hyperactive, was 46.2%. However, the indices of the model selection in our study was a 3-class model rather than the 5-class model [20]. The features of internalizing behavior in our moderate group were close to the normal group, while the features of externalizing behavior were high probability of “Hyperactivity/Inattention” with the characteristics of usually lying, did not follow the request from adults, and was not liked by other children. The SDQ is frequently used as a screening and outcome measurement tool for attention deficit hyperactivity disorder (ADHD) in clinical and research settings [40]. Children in this group may have some pre-symptoms of ADHD. Since “Hyperactivity/Inattention” co-occurred with “did not liked by other children”, it can be explained that children with inattention, excessive motor activity, and impulsivity commonly engaged in inappropriate social behavior including bothering others and becoming involved in uninvited conversations. This may make them become less socially preferred, easily rejected by their peers, and finally suffer from a lack of friends [41,42]. Additionally, we also found that “Hyperactivity/Inattention” co-occurred with “often lies and cheats.” This can be explained by the following: (1) Children with impulsivity are unable to stop and think before they act. Thus, they are more likely to do things that get them in trouble, and then lie about it [43]. (2) Children with ADHD symptoms overestimate their competence [44]. Their thoughts will be overly optimistic and unrealistic and they will tell others about their wishful thinking [43], which may also result in ignoring adults’ requests. (3) Hyperactivity/inattention symptoms are negatively related to the school performance of children [45], heating and lying, please their parents, teachers, and other adults in their lives, and blind their eyes [43]. 

The lowest probability (23.8%) group was the normal group, which showed relatively stable and fewer internalizing and externalizing behavioral problems. This group was also similar to the Chinese left-behind children’s study (27%) [19], and Spanish children’s study (19.7%) [20], but less than that of Chinese adolescents (49.1%) [4]. 

With these results, our 3-class LCA model using a Japanese suburban community-based sample was closest to the Chinese left-behind children’s sample, which was categorized as a 3-class model. However, the probabilities of these three classes identified in China were lower than 40%, even in the high-risk group. This can be explained by the fact that the sample included primary school, middle school, and high school children, while our sample only included primary school children. The middle and high school children in the Chinese study may have resulted in a decrease in the prevalence of both behavioral problems, because the prevalence of primary school children in the high school group may be higher than adolescents.

Lastly, we also found that family activities such as playing sports, traveling or hiking, watching TV and communicating, cooking or making a dessert, and doing housework were related to latent class memberships according to children’s internalizing and externalizing behaviors. A diversity of family-based group tasks and activities can improve family communication, harmony, and cohesion [46]. Higher family cohesion is related to a lower incidence of both behaviors [47]. Higher family cohesion can establish a positive emotional connection and secure relationships between caregivers and children [48]. Children are encouraged by this favorable family relationship to express their feelings directly, which may make parents notice and deal effectively with behavioral and emotional problems at an early stage [48,49]. Additionally, family activities brought more opportunities for children to have human and social stimulation, which may also affect their emotions and behaviors [24,25]. However, we did not find any significant results in the relationship between eating outside, going to cinema or concert, going shopping, playing indoor games, and each latent class membership. This may be explained by the following: (1) we did not consider the frequency and quality of these family activities; (2) activities such as eating outside and going to cinema or concert may not allow children to communicate with their caregivers since parents teach their children to be polite and to keep silent in public spaces. As for shopping and playing indoor games, a previous study showed that children who go shopping with their parents more than one to three times a month will have better social competence and vocabulary skills [50]. Additionally, parents playing with their children more than one to three times a month will also help them achieve better social competence and intelligence development [25]. Thus, the frequency and time duration that we did not consider in this study may have caused biases.

To the best of our knowledge, the present study is significant because this is the first attempt to use the LCA method to identify the subtyping internalizing and externalizing behaviors among Japanese suburban community-based samples. Second, this was a community-based survey with a high data-utilization rate. All families with primary students in the community were recruited; thus, there was no selection bias. Considering that this community is a typical community in Japan [24], we considered that the results may be generalizable to other rural areas in Japan. Third, previous studies only examined the relationship between age, gender, siblings, and latent class memberships. Meanwhile, this study focused on the relationship between different types of family activities and latent class memberships, which provides evidence of activities that may be used to conduct the prevention section. 

The limitations of this study need to be considered. First, the causal relationship could not be explained because this was a cross-sectional study. Second, the large no-answer rate, some demographic information, including the parents’ educational background and family’s economic status, could not be considered in the analyses. Third, there is a possibility of a recall bias as self-report questionnaires were used, and the study only considered the types and frequencies of family activities, while the quality of these activities was not considered. Thus, as an implication of the present study, we hope that future studies can focus on exploring the potential growth model under a longitudinal design regarding the internalizing and externalizing behaviors of children. For example, some children may fall into the category of increasing externalization behavior, while others may fall into the category of increasing externalization behavior and then decreasing it. Understanding the potential types of changes in children’s emotional and behavioral problems can help us to design prompt support plans before the worst of the symptoms are displayed. Additionally, the longitudinal effects of family variables on latent class memberships need to be confirmed in the future. Although this study focused on schoolchildren, there is a paucity of data on the internalizing and externalizing behaviors among preschool children, using mixed models such as latent class analysis or the latent transition analysis. Thus, studies on children in the early age ranges are needed. Lastly, as the findings of this study cannot be generalized to other countries, distinctive groups should also be identified in more countries with different culture backgrounds.

## 5. Conclusions

This study explored the potential types related to children’s internalizing and externalizing behaviors among Japanese suburban community-based samples. Three groups were highlighted, namely, risk, moderate, and normal. We also examined the relationship between different types of family activities and latent class memberships of children’s internalizing and externalizing behaviors. We found that frequent family activities, such as playing sports, traveling or hiking, watching TV and communicating, cooking or making a dessert, and doing housework were significantly related to the normal group. Our results will be useful in improving the understanding of potential comorbidities of both behaviors, as well as their link to family activities. We recommend that when related institutions in the community establish support plans for schoolchildren undergoing emotional and behavioral problems, they consider categorizing the schoolchildren into the three potential groups identified in this study, thereby making the support plan more targeted. Besides, as this study has identified several activities to improve behavior, support institutions can consider encouraging families to perform them together. As for the families, parents can be encouraged to undertake activities, such as completing housework with their children. Health-related professionals and health policy makers can consider providing support for regular activities such as family sports competitions and family traveling events to inspire families’ engagement.

## Figures and Tables

**Table 1:** Participant characteristics (N = 206).

Variable	Category	N	%
Age of children	6–7	68	33.0
	8–9	72	35.0
	10–11	66	32.0
Gender of children	Boy	109	52.9
	Girl	97	47.1
Family structure	Nuclear (Parents, children)	106	51.5
	Extend (Grandparents, parents, and children)	100	48.5
Siblings	Sibling	174	84.5
	No sibling	32	15.5
Disease	No	189	91.7
	Yes	17	8.3
Eat outside	Yes	128	62.1
	No	78	37.9
Play sports	Yes	59	28.6
	No	147	71.4
Traveling or hiking	Yes	56	27.2
	No	150	72.8
Go to cinema or concert	Yes	36	17.5
	No	170	82.5
Go shopping	Yes	145	70.4
	No	61	29.6
Play indoor games	Yes	70	34.0
	No	136	66.0
Watch TV and communicate	Yes	111	53.9
	No	95	46.1
Cook or make a dessert	Yes	43	20.9
	No	163	79.1
Do housework	Yes	82	39.8
	No	124	60.2

**Table 2:** Participant characteristics (N = 206).

Model	Log-Likelihood	G-Squared	AIC	BIC	CAIC	aBIC	Entropy	df
2 class	−2140.26	2138.48	2220.48	2356.92	2397.92	2227.02	0.82	1,048,534
3 class	−2083.92	2025.79	2149.79	2356.12	2418.12	2159.68	0.82	1,048,513
4 class	−2039.54	1937.04	2103.04	2379.25	2462.25	2116.27	0.87	1,048,492
5 class	−2013.13	1884.22	2092.22	2438.32	2542.32	2108.81	0.88	1,048,471
6 class	−2005.28	1868.52	2118.52	2534.51	2659.51	2138.46	0.89	1,048,450

**Table 3:** The probability of the 3 class model. (N = 206).

Category	Negative Response Probabilities (%)		
	Class 1 Risk Group (N = 67)	Class 2 Moderate Group (N = 90)	Class 3 Normal Group (N = 49)
Often complains of headaches	43.9	22.3	14.3
Worry a lot	50.0	6.9	2.8
Unhappy, depressed	61.7	13.4	8.2
Nervous in new situation	83.8	26.5	7.0
Many fears, easily scared	76.4	25.0	15.5
Would rather be alone	48.2	34.4	17.9
Have one good friend	31.5	19.1	8.7
Other children bully me	45.8	16.3	2.9
Get along better with adults	56.9	19.0	18.2
Other people like me	74.6	63.8	30.4
Often lose my temper	84.7	41.6	35.5
Usually do as I am told	85.7	74.1	43.3
Fight a lot	27.4	4.8	4.1
Accused of lying or cheating	66.6	39.9	0.4
Take things that are not mine	13.9	0.0	0.0
Restless, overactive	72.2	47.9	4.4
Fidgeting or squirming	45.1	12.2	0.1
Easily distracted	81.7	51.8	0.5
Thinks things out before acting	97.9	89.7	46.8
Sees tasks through to end	86.9	83.7	15.5

**Table 4:** Chi-square results for the association between the family activities and children’s internalizing and externalizing behaviors. (N = 206).

Item	Category	Total	Risk		Moderate		Normal		*p*
			N	%	N	%	N	%	
Eat outside	Yes	128	45	35.1	55	43.0	28	21.9	0.527
	No	78	22	28.2	35	44.9	21	26.9	
Play sports	Yes	59	15	25.4	23	39.0	21	35.6	0.038
	No	147	52	35.4	67	45.6	28	19.0	
Traveling or hiking	Yes	56	10	17.9	31	55.4	15	26.8	0.021
	No	150	57	38.0	59	39.3	34	22.7	
Go to cinema or concert	Yes	36	7	19.4	18	50.0	11	30.6	0.171
	No	170	60	35.3	72	42.2	38	22.4	
Go shopping	Yes	145	51	35.2	61	42.0	33	22.8	0.457
	No	61	16	26.2	29	47.6	16	26.2	
Play indoor games	Yes	70	22	31.4	29	41.4	19	27.2	0.717
	No	136	45	33.1	61	44.9	30	22.0	
Watch TV and communicate	Yes	111	26	23.4	54	48.6	31	28.0	0.010
	No	95	41	43.2	36	37.9	18	18.9	
Cook or make a dessert	Yes	43	14	32.6	12	27.9	17	39.5	0.012
	No	163	53	32.5	78	47.9	32	19.6	
Do housework	Yes	82	18	22.0	39	47.6	25	30.4	0.021
	No	124	49	39.5	51	41.1	24	19.4	
Age	6–7	68	30	44.1	26	38.2	12	17.6	0.042
	8–9	72	24	33.3	32	44.4	16	22.2	
	10–11	66	13	19.7	32	48.5	21	31.8	
Gender	Boy	109	33	30.3	54	49.5	22	20.2	0.179
	Girl	97	34	35.1	36	37.1	27	27.8	
Family structure	Nuclear	106	38	35.8	48	45.3	20	18.9	0.213
	Extend	100	29	29.0	42	42.0	29	29.0	
Siblings	Sibling	174	51	29.3	76	43.7	47	27.0	0.015
	No sibling	32	16	50.0	14	43.8	2	6.3	
Disease	No	189	59	31.2	84	44.4	46	24.3	0.407
	Yes	17	8	47.1	6	35.3	3	17.6	

**Table 5:** Participant characteristics (N = 206).

Variables	Group	OR	95% CI	*p*
Play sports	Normal	Ref.	Ref.	Ref.
	Moderate	0.38	0.18–0.83	0.015
	Risk	0.27	0.11–0.65	0.004
Traveling or hiking	Normal	Ref.	Ref.	Ref.
	Moderate	1.07	0.50–2.29	0.861
	Risk	0.32	0.12–0.83	0.019
Watch TV and communicate	Normal	Ref.	Ref.	Ref.
	Moderate	0.83	0.40–1.70	0.602
	Risk	0.33	0.15–0.74	0.007
Cook or make a dessert	Normal	Ref.	Ref.	Ref.
	Moderate	0.32	0.13–0.74	0.008
	Risk	0.23	0.25–1.41	0.591
Do housework	Normal	Ref.	Ref.	Ref.
	Moderate	0.73	0.36–1.48	0.387
	Risk	0.34	0.15–0.77	0.010

Note: Adjusted with age and siblings.

## Data Availability

Not applicable.
